# High prevalence of MASLD in psoriasis and psoriatic arthritis assessed with multiparametric magnetic resonance imaging

**DOI:** 10.1093/rheumatology/keaf344

**Published:** 2025-06-26

**Authors:** Lija James, Charlie Diamond, Hussein Al-Mossawi, Anneli Andersson, Prashant Pandya, Elizabeth Shumbayawonda, Leila Izadi Firouzabadi, Lily Watson, Laura J Savage, Helena Thomaides-Brears, Rajarshi Banerjee, Laura C Coates

**Affiliations:** Nuffield Department of Orthopaedics, Rheumatology and Musculoskeletal Sciences, University of Oxford, Oxford, UK; Perspectum Ltd, Oxford, UK; Nuffield Department of Orthopaedics, Rheumatology and Musculoskeletal Sciences, University of Oxford, Oxford, UK; Perspectum Ltd, Oxford, UK; Perspectum Ltd, Oxford, UK; Perspectum Ltd, Oxford, UK; Dermatology Department, Churchill Hospital, Oxford University Hospitals NHS Trust, Oxford, UK; University of Oxford, Oxford, UK; Leeds Centre for Dermatology, Chapel Allerton Hospital Department of Dermatology, Leeds Teaching Hospitals NHS Trust, Leeds, UK; Perspectum Ltd, Oxford, UK; Perspectum Ltd, Oxford, UK; Nuffield Department of Orthopaedics, Rheumatology and Musculoskeletal Sciences, University of Oxford, Oxford, UK

**Keywords:** psoriatic arthritis, psoriasis, MRI, MASLD, MASH, methotrexate, cT1

## Abstract

**Objectives:**

Psoriatic disease (PsD) is a chronic inflammatory condition associated with obesity, metabolic dysfunction-associated steatotic liver disease (MASLD) and steatohepatitis (MASH). We aimed to determine the prevalence of MASLD/MASH in a real-world psoriatic cohort using advanced imaging.

**Methods:**

COLIPSO is a multicentre, prospective study of adults with PsD receiving standard systemic therapy in secondary care. Fifty participants underwent clinical assessments and non-invasive tests of liver health, including MRI-biomarkers of liver fibro-inflammation and fat content. These were compared with 360 individuals with PsD, with 150 controls without PsD (matched for age, sex, BMI and comorbidities) and 150 healthy controls from the UK Biobank and COVERSCAN studies. Associations were investigated with Spearman’s rank correlations and multivariate linear regression models.

**Results:**

The prevalence of steatotic liver disease was 44% in the PsD group (aged 48 years, 58% male, BMI 29 kg/m^2^) and higher than in matched controls (25%, *P* = 0.02). MASH was prevalent in 22% of PsD patients (*vs* 3% in matched controls, *P* < 0.001). Thirteen of the 24 (54%) individuals with PsD and liver disease had normal liver function blood tests. No significant difference in levels of liver disease was observed between those with or without prior exposure to methotrexate.

**Conclusions:**

Individuals with PsD exhibit higher prevalence of MASLD and MASH that was missed by blood tests and was present even in individuals with no methotrexate exposure. These findings underscore the importance of routine MASLD screening in this population with more sensitive tools, such as multi parametric MRI.

## Graphical Abstract

**Figure:**
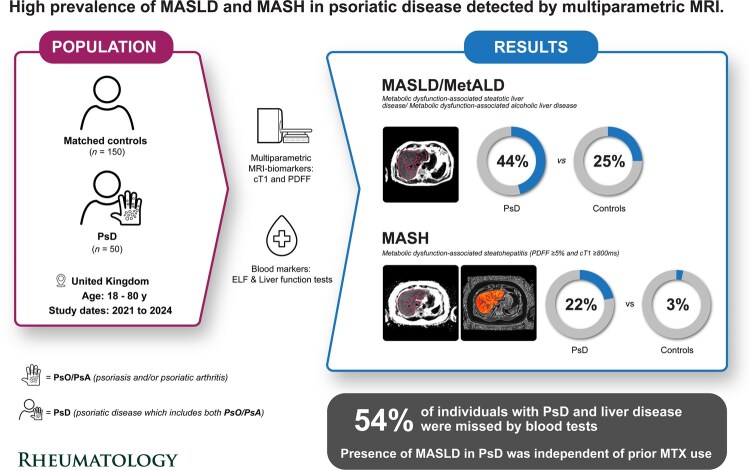

Rheumatology key messages

- PsD patients have more metabolic liver disease compared with controls with similar BMI and comorbidities.
- Liver disease is seen even in patients with no previous exposure to methotrexate (liver fibro-inflammation/fat).
- Blood tests miss half of metabolic liver disease detected by multiparametric MRI in PsD patients.

## Introduction

Psoriatic disease (PsD) is a systemic inflammatory disorder that includes psoriasis (PsO), presenting with distinct cutaneous signs (well circumscribed erythema, induration and silvery-scale with or without nail changes), and psoriatic arthritis (PsA), a type of arthritis that can lead to irreversible joint damage and disability. Affecting 1–8% [1] of adults worldwide, PsD frequently coexists with metabolic comorbidities such as obesity, type 2 diabetes mellitus and metabolic syndrome [2]. Among these, metabolic dysfunction-associated steatotic liver disease (MASLD) and metabolic dysfunction-associated alcoholic liver disease (MetALD), characterized by increased fat in hepatocytes, remain the least studied. There is a notable lack of evidence on coexistence of these liver diseases in PsA overall, and in PsO (without PsA) using reliable methods of diagnosis.

In clinical practice patients with PsD are not routinely screened or monitored for MASLD, despite existing literature indicating a higher prevalence of MASLD in individuals with coexisting PsO [2–6]. Methotrexate (MTX), routinely the first-line therapy for PsD, is thought to carry risk of hepatotoxicity [7–10]. MASLD is often an incidental finding during routine monitoring for hepatotoxicity in PsD patients on systemic treatment. Without management, MASLD can progress to metabolic dysfunction-associated steatohepatitis (MASH), characterized by liver inflammation and hepatocyte ballooning, as well as steatosis, and elevated risk of progression to liver fibrosis and cirrhosis [11]. The current reference standard for diagnosis of MASH is liver biopsy, which is invasive, high cost, and associated with risk of major complications including long recovery time and sampling limitations [12]. There is a progressive shift to non-invasive testing for MASLD/MASH. These tests include serum biomarkers for liver function, such as alanine aminotransferase (ALT) and aspartate aminotransferase, and for liver fibrosis, such as the enhanced liver fibrosis (ELF) test. Imaging tools are also commonly used for diagnosis and prognosis of MASLD, such as ultrasound-assisted vibration-controlled transient elastography (VCTE) and magnetic resonance imaging (MRI) [13]. The utility of ELF has not been evaluated in PsD populations and ultrasound has technical limitations, particularly in patients with obesity [14–17].

Recent advancements in MRI enable more accurate evaluation of liver disease. The reference non-invasive method for steatosis staging is multiparametric MRI (liver fat content with proton density fat fraction) validated against steatosis grades on biopsy [18, 19]. Liver inflammation and fibrosis can be quantified by the MRI metric corrected T1 (cT1) [20]. Elevated cT1 values indicate presence of liver fibro-inflammation in MASH [21–23] and autoimmune hepatitis [24, 25], and predict liver- and cardiovascular-related outcomes [26–28]. Use of multiparametric MRI that includes cT1 and liver fat metrics (LiverMultiScan) has been shown to be cost-effective in the European pathway for MASLD care but has not been assessed in PsD before [29].

The primary objective of this study was to determine the prevalence of steatotic liver disease and fibro-inflammation in individuals with PsD under standard of care, using multiparametric MRI. We therefore compared a prospective cohort of individuals with PsD from secondary care and a retrospective group with PsD from the UK general population (UK Biobank) to individuals without PsD (fully matched controls and healthy controls). We also evaluated the association of liver disease with methotrexate use and severity of PsD.

## Methods

### Study design and participants

#### COLIPSO study participants from secondary care clinics

The Co-Prevalence of Liver Disease in Psoriatic Disease (COLIPSO) study (London—Bromley Research Ethics Committee, REC: 20/LO/0616) is a real-world, multicentre study adopting a prospective, observational, cohort study design in individuals with PsD. All patients provided written informed consent. Adults aged ≥18 years old with PsD were recruited from the rheumatology and dermatology clinics at Oxford University Hospitals NHS Foundation Trust and Leeds Teaching Hospitals NHS Trust between 2021 and 2024 (Supplementary Fig. S1, available at *Rheumatology* online). The participants were eligible if they had moderate to severe PsO or PsA confirmed by their consultant dermatologist or rheumatologist and starting a new systemic treatment with conventional synthetic or biologic DMARD as part of their standard care. Participants with PsO alone were eligible for inclusion only if they had no clinical evidence of joint disease at the time of recruitment. Exclusion criteria included any contraindications to MRI, a diagnosis of autoimmune hepatitis, viral hepatitis, Wilson’s disease, or other significant disease that, in the investigator’s opinion, could pose a risk for study participation or completion.

#### Comparison groups

To provide a comprehensive evaluation of liver disease prevalence in PsD patients, we compared this prospective PsD cohort with three additional groups of individuals from other studies using retrospective data (selected from UK Biobank [application 9914] [27, 28] or COVERSCAN clinical trial [registration number: NCT04369807] [30], as appropriate; Supplementary Data S1, available at *Rheumatology* online). The comparison groups were the following: (i) matched controls: 150 individuals without PsD fully matched to cases from the prospective COLIPSO cohort, by age, sex, BMI, hypertension, hyperlipidaemia and diabetes status; (ii) healthy controls: 150 healthy controls without PsD and without obesity matched by age and sex only; and (iii) patients with PsD from general population: 360 people with PsD, identified by International Classification of Diseases, 10th Revision (ICD-10 codes) from the UK Biobank (Supplementary Data S1, available at *Rheumatology* online).

### Data collection

#### Clinical and biochemical analysis

For the COLIPSO cohort, data were collected on demographics, anthropometric measurements, medical history, alcohol intake, disease duration, Psoriasis Area and Severity Index (PASI) scores, percentage body surface area (%BSA) affected by psoriasis, prior and current use of DMARDs, liver function tests (bilirubin, albumin, ALT or alkaline phosphatase), ELF scores, CRP levels and patient self-reported outcomes related to health-related quality of life (Supplementary Data S2, available at *Rheumatology* online). For PsA individuals specifically, Disease Activity in Psoriatic Arthritis (DAPSA) scores were collected, based on 68 tender and 66 swollen joint counts, individual reported pain scores and CRP levels. A PASI score of ≥10 defined moderate-to-severe PsD and a DAPSA score of ≥28 defined severe PsA [31].

All comparison groups from the general population had demographic, anthropometric and MRI data, but no relevant serological or biochemical data were available.

#### MRI acquisition and analysis

All individuals had a standardized multiparametric MRI scan (LiverMultiScan, Perspectum, Oxford), which lasted ∼10 min with methods previously demonstrated [32, 33]. All quantitative MRI methods were deployed on standard clinical MRI scanners (GE Signa Voyager 1.5 T (Chicago, IL, USA), Siemens Area 1.5 T (Forchheim, Germany) and Philips Achieva dStream 3 T (Amsterdam, Netherlands)), and data acquired and processed by trained MR technologists and radiographers blinded to all clinical data. Data were centrally curated and quality controlled.

#### Definitions of liver MRI metrics

Normal values/reference ranges for MRI liver fat content and fibro-inflammation were defined previously at <5% and <800 ms, respectively [21, 34]. Terminology and diagnostic criteria for MASLD and metabolic-dysfunction-associated alcoholic liver disease (MetALD; previously non-alcoholic fatty liver disease, NAFLD) are adopted according to international consensus [35]. MASH was defined by both elevation in liver fat content (≥5%) and fibro-inflammation (cT1 ≥ 800ms) and a cardiometabolic risk factor. These thresholds have previously been shown to be diagnostic of steatohepatitis in biopsy-paired datasets, with cT1 ≥ 875ms associated with risk of significant liver fibrosis [21]. Advanced MASH was here defined as cT1 ≥ 875 ms, liver fat ≥5% and ≥1 cardiometabolic risk factor.

### Statistical analysis

The study was powered for the primary end point of detecting a significant difference in the prevalence of elevated liver fibro-inflammation (cT1 ≥ 800ms) between individuals with PsD and fully matched controls without PsD, based on a two-sample proportion test with 90% power and α of 0.05. At the final sample size of *n* = 50 PsD with a predicted prevalence of 30% and *n* = 150 controls, this enabled a minimum detectable prevalence difference of 23%.

The descriptive statistics for continuous and categorical variables are expressed with the mean (s.d.) and frequency (percentage prevalence), respectively. For groupwise comparisons, Wilcoxon’s rank-sum test was used for continuous variables, and Fisher’s exact test was used for categorical variables. Spearman’s rank correlation coefficients were calculated to assess associations, while multivariate linear regression models were used to adjust for confounding variables. Statistical significance was defined by a *P*-value threshold of <0.05 (two-sided). All statistical analyses were conducted in R software version 4.2.1 (R Foundation for Statistical Computing, Vienna, Austria).

## Results

### Study populations

#### People with PsD from secondary care

Fifty individuals with PsD (34 with PsA and 16 with PsO) were recruited from secondary care rheumatology or dermatology clinics between 2021 and 2024. Their mean age was 48 years, 58% were male, with mean BMI of 29 kg/m^2^. Known comorbidities were obesity (32%), hypertension (20%) and hyperlipidaemia (18%), with type 2 diabetes in only 2% (Table 1). The mean duration of psoriatic symptoms was 19 years (s.d. 15), with 28% having moderate or severe PASI scores. Among the 34 PsA individuals, the duration of disease was ≥10 years in 16/34 (47%), and 16/34 (47%) had a high DAPSA score. MTX use was common; 27% of individuals were currently on MTX and 41% had been treated with MTX during a mean 20-year (s.d. 15) disease course (Supplementary Table S1, available at *Rheumatology* online). Total cumulative dose of MTX was unavailable. The participants underwent an evaluation of the liver by multiparametric MRI, with a median time of 26 days (interquartile range 12–40) between clinical and imaging visit.

**Table 1.** Baseline demographics

	PsD from secondary care (COLIPSO) (*n* = 50)	Matched controls without PsD (*n* = 150)	*P*-value, PsD *vs* matched controls	Healthy controls (*n* = 150)	*P*-value, PsD *vs* healthy controls	PsD in general population (UKBB) (*n* = 360)	*P*-value, PsD in COLIPSO *vs* PsD in UKBB
Demographics							
Age, mean (s.d.), years	48 (14)	50 (13)	0.216	49 (13)	0.407	67 (8)	**<0.001**
Sex (male), *n* (%)	29 (58)	87 (58)	>0.999	91 (61)	0.742	200 (56)	0.764
BMI, mean (s.d.), kg/m^2^	29 (5)	27 (5)	0.122	24 (3)	**<0.001**	28 (5)	0.291
Categories, *n* (%)			0.225		**<0.001**		0.368
Lean (<25 kg/m^2^)	10 (20)	49 (33)		89 (59)		107 (30)	
Overweight (≥25–<30 kg/m^2^)	24 (48)	57 (38)		61 (41)		150 (42)	
Obese (≥30 kg/m^2^)	16 (32)	44 (29)		0 (0)		103 (29)	
PsA, *n* (%)	34 (68)	—	—	—	—	94 (26)	**<0.001**
PsO, *n* (%)	16 (32)	—	—	—	—	291 (81)	**<0.001**
Type 2 diabetes, *n* (%)	1 (2)	4 (3)	>0.999	0 (0)	0.25	45 (13)	**0.029**
Type 1 diabetes, *n* (%)	2 (4)	16 (11)	0.252	1 (1)	0.155	4 (1)	0.159
Hypertension, *n* (%)	10 (20)	38 (25)	0.567	21 (14)	0.367	191 (53)	**<0.001**
Hyperlipidaemia, *n* (%)	9 (18)	43 (29)	0.192	9 (6)	**0.019**	156 (43)	**<0.001**
Cardiometabolic risk factor, *n* (%)	43 (86)	111 (74)	0.119	71 (47)	**<0.001**	308 (68)	>0.999
High alcohol use, *n* (%)	3 (6)	9 (6)	>0.999	6 (4)	0.693	63 (18)	**0.039**
Liver MRI metrics							
Liver fat content—PDFF (%), mean (s.d.)	7 (7)	5 (4)	0.259	3 (2)	**0.007**	6 (6)	0.496
PDFF ≥ 5%, *n* (%)	24 (48)	41 (28)	**0.014**	22 (15)	**<0.001**	155 (43)	0.545
MASLD/MetALD^a^, *n* (%)	22 (44)	38 (25)	**0.02**	18 (12)	**<0.001**	150 (42)	0.762
Liver fibro-inflammation—cT1, mean (s.d.), ms	790 (138)	711 (54)	**<0.001**	694 (49)	**<0.001**	726 (56)	**<0.001**
cT1 ≥ 800 ms, *n* (%)	15 (30)	10 (7)	**<0.001**	5 (3)	**<0.001**	39 (11)	**<0.001**
cT1 ≥ 875 ms, *n* (%)	9 (18)	0 (0)	**<0.001**	0 (0)	**<0.001**	4 (1)	**<0.001**
Steatohepatitis^b^, *n* (%)	12 (24)	5 (3)	**<0.001**	0 (0)	**<0.001**	36 (10)	**0.008**
MASH^c^, *n* (%)	11 (22)	5 (3)	**<0.001**	0 (0)	**<0.001**	35 (10)	**0.016**
Severe MASH^d^, *n* (%)	8 (16)	0 (0)	**<0.001**	0 (0)	**<0.001**	4 (1)	**<0.001**

Demographic data comparing PsD from prospective COLIPSO cohort *vs* matched controls without PsD, healthy controls and individuals with PsD from the general population (UK Biobank).

a PDFF ≥ 5% and cardiometabolic risk factor.

b cT1 ≥ 800 ms and PDFF ≥ 5%.

c cT1 ≥ 800 ms and PDFF ≥ 5% and cardiometabolic risk factor.

d cT1 ≥ 875 ms and PDFF ≥ 5% and cardiometabolic risk factor. Text in bold indicate statistical significance, *P* ≤ 0.05. cT1: corrected T1; MASH: metabolic dysfunction-associated steatohepatitis; MASLD: metabolic dysfunction-associated steatotic liver disease; MetALD: metabolic dysfunction-associated alcoholic liver disease; PDFF: proton density fat fraction; PsA: psoriatic arthritis; PsD: psoriatic disease; PsO: psoriasis.

#### Matched controls without PsD and healthy controls

The 150 matched individuals without PsD were aged 50 years, 58% male, mean BMI of 27 kg/m^2^, 29% obese, 25% hypertensive, 29% with hyperlipidaemia and 3% with type 2 diabetes. The 150 healthy controls were aged 49 years, 61% male, mean BMI of 24 kg/m^2^, none were obese, 14% were hypertensive and 6% had hyperlipidaemia (Table 1).

#### People with PsD from the UK general population

These 360 individuals with PsD, mainly PsO (81%), were older and had a shorter PsD duration but a higher prevalence of comorbidities, despite having a similar BMI, when comparing with the people with PsD from secondary care (Table 1). They were aged 67 years, 56% male, mean BMI of 28 kg/m^2^, 29% obese, 13% with type 2 diabetes, 53% were hypertensive, 43% with hyperlipidaemia and an average PsD duration of 7 (s.d. 6) years.

### Prevalence of MASLD/MetALD from imaging

**Figure 1.**
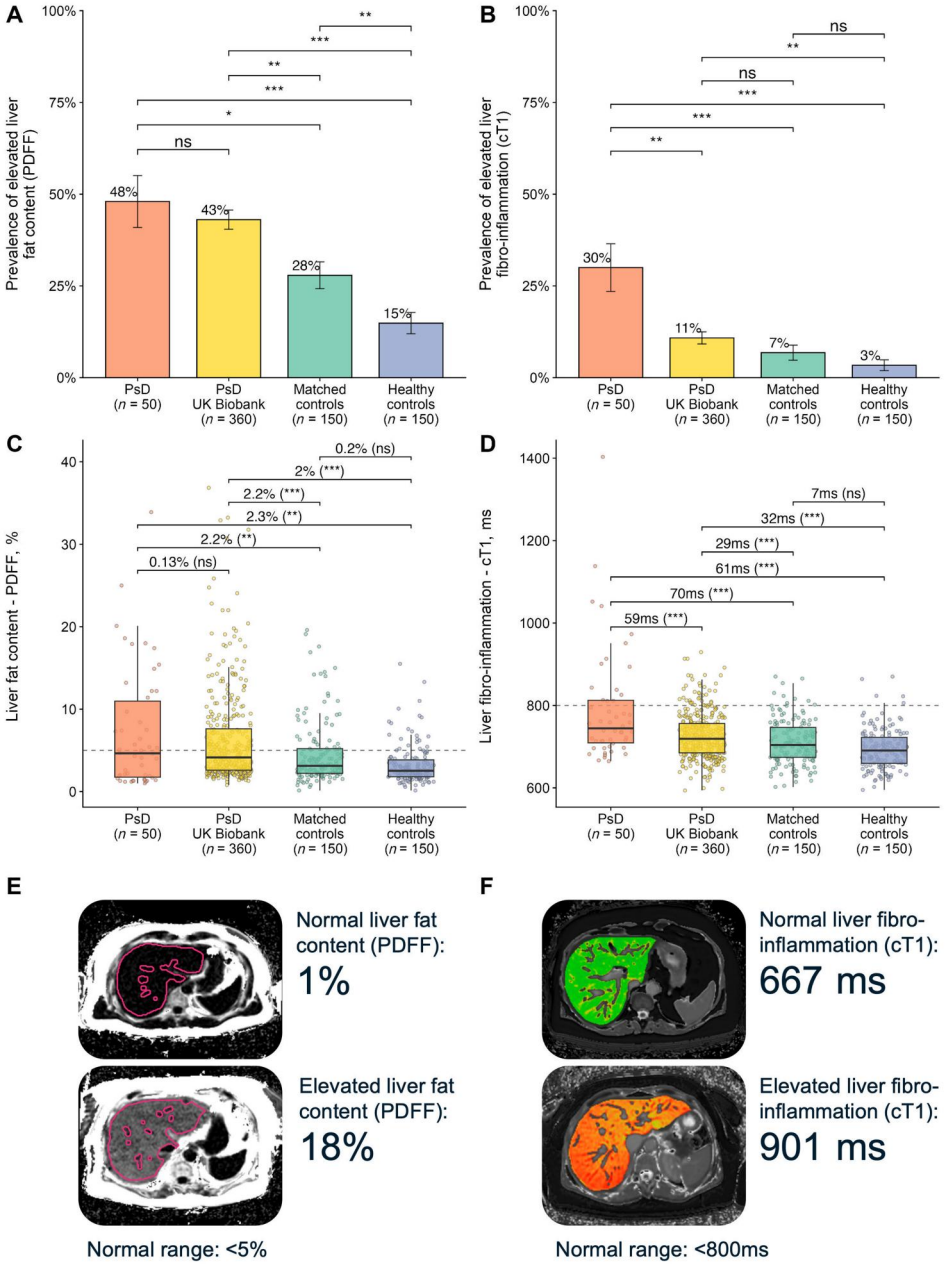
MASLD in psoriatic disease. (**A**, **B**) Prevalence of elevated liver fat (**A**) and fibro-inflammation (**B**) in PsD patients from the COLIPSO cohort and comparator groups. (**C**, **D**) Distribution of liver fat content (**C**) and fibro-inflammation (**D**) across all study populations, with pairwise comparisons adjusted for age, sex and BMI using multivariate linear regression. (**E**, **F**) Example images by multiparametric MRI of PsD individuals with normal and elevated liver fat content (**E**) and fibro-inflammation (**F**). *P < 0.05, **P < 0.01, ***P < 0.001; ns: not significant. cT1: corrected T1; MASLD: metabolic dysfunction-associated steatotic liver disease; PDFF: proton density fat fraction; PsD: psoriatic disease

In the PsD cohort from secondary care, 24/50 patients (48%) showed liver steatosis, and this was equally prevalent in both PsO and PsA (*P* = 0.55, Supplementary Table S1, available at *Rheumatology* online). This disease was classified as MASLD in 19 (38%) and as MetALD in three (6%). Notably, elevated liver fat was found in two individuals with normal BMI, no cardiometabolic risk factors nor high alcohol intake. In the people with PsD from the general population, MASLD was similarly prevalent (in 117/360, 33%, *P* = 0.43), as was MetALD (33/360, 9%, *P* = 0.60) (Table 1, Fig. 1).

In contrast, MASLD/MetALD was significantly less prevalent in matched controls without PsD (38/150, 25%, *P* = 0.02, all but three were MASLD). In the healthy controls without obesity MASLD was even rarer (18/150, 12%, *P* < 0.001, and there were no MetALD) (Fig. 1).

### Steatohepatitis from imaging

Individuals with PsD from secondary care and the general population exhibited higher mean levels of liver fibro-inflammation, as measured by cT1, compared with matched controls without PsD and healthy controls without obesity (790 ms [s.d. 138] and 726 ms [s.d. 56] *vs* 711 ms [s.d. 54] and 694 ms [s.d. 49], respectively, *P* < 0.001). Elevated liver fibro-inflammation was similarly prevalent in both PsO and PsA (*P* = 0.51, Supplementary Table S1, available at *Rheumatology* online).

MASH was defined based on the presence of both MetALD/MASLD and elevated cT1. The prevalence of MASH was 22% (11/50) in individuals with PsD from secondary care and 10% (36/360) in those from the general population. In contrast, MASH was rare (3%, *P* < 0.001) in matched controls without PsD and absent in the healthy controls without obesity.

Additionally, 16% (8/50) individuals with PsD from secondary care had advanced MASH with higher levels of fibro-inflammation, while none of the matched controls without PsD met those criteria (0%, *P* < 0.001), Table 1.

### Liver fibro-inflammation without liver fat

Three individuals with PsD showed elevated liver fibro-inflammation without high liver fat. These individuals did not exhibit distinguishing characteristics in terms of demographics or treatment history; however, all three had at least one cardiometabolic risk factor. We therefore investigated whether liver fibro-inflammation was associated with PsD independently of liver fat. In a multivariate model adjusting for liver fat content and demographic differences, individuals with PsD still exhibited significantly greater mean levels of liver fibro-inflammation compared with matched controls (+43 ms [95% CI: 23–64 ms]).

### Serological and clinical associations with liver disease

#### Liver non-invasive blood markers in individuals with PsD from secondary care

In individuals with PsD and high liver fat, 13/24 (54%) showed normal liver function or no fibrosis in serological tests (Supplementary Tables S2 and S3, available at *Rheumatology* online). Two of the three people with MetALD showed elevation in ALT, of which one also had abnormal ELF and advanced disease on imaging (cT1 > 875 ms). Elevation in ALT was detected in 5/19 (26%) individuals with coexisting PsD and MASLD. In the 11 individuals with PsD and MASH from imaging, only 3/11 (27%) also had abnormal ELF alongside elevated ALT. These three individuals also represented the 3/8 individuals with advanced MASH.

ALT was positively correlated with liver fat content (rho = 0.44, *P* < 0.001) but not with liver cT1. ELF did not correlate with either cT1 (rho = 0.13, *P* = 0.39) or liver fat content (rho = 0.23, *P* = 0.12).

#### Association of liver disease with PsD severity

We found no significant demographic, biochemical or imaging differences between PsA and PsO individuals. Individuals with PsO had a higher mean ALT but the prevalence of clinically elevated ALT was similar between disease groups. Health-related quality of life was found to be poorer in PsA than PsO, particularly in terms of physical functioning, pain and general health (Supplementary Table S1, available at *Rheumatology* online).

**Figure 2.**
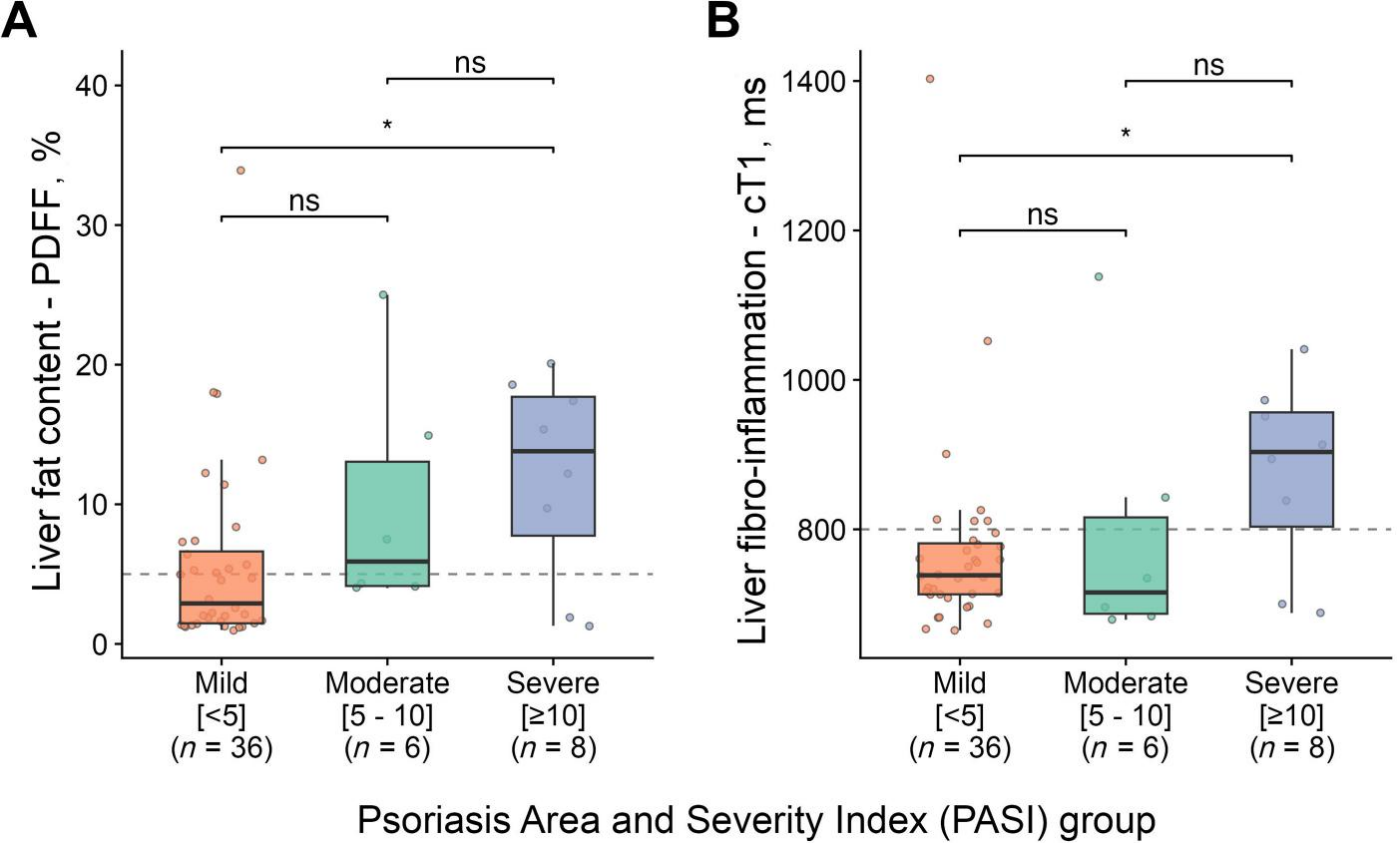
The impact of psoriasis disease activity on liver health. The figure shows (**A**) distribution of liver fat content, measured by PDFF (%) and (**B**) liver fibro-inflammation (cT1) by PASI score severity. *P < 0.05; ns: not significant. cT1: corrected T1; PASI: Psoriasis Area and Severity Index; PDFF: proton density fat fraction

Both mean liver fat content and liver fibro-inflammation by cT1 were significantly higher in individuals with severe PASI scores (Fig. 2). Liver cT1 correlated with PASI scores in individuals with PsO (rho = 0.58, *P* = 0.02), but there was no correlation with DAPSA in individuals with PsA. Liver fat content did not correlate with any measure of psoriatic disease clinical severity, irrespective of disease type (PsA or PsO) (Supplementary Table S4, available at *Rheumatology* online).

#### Association of liver disease with MTX treatment

**Figure 3.**
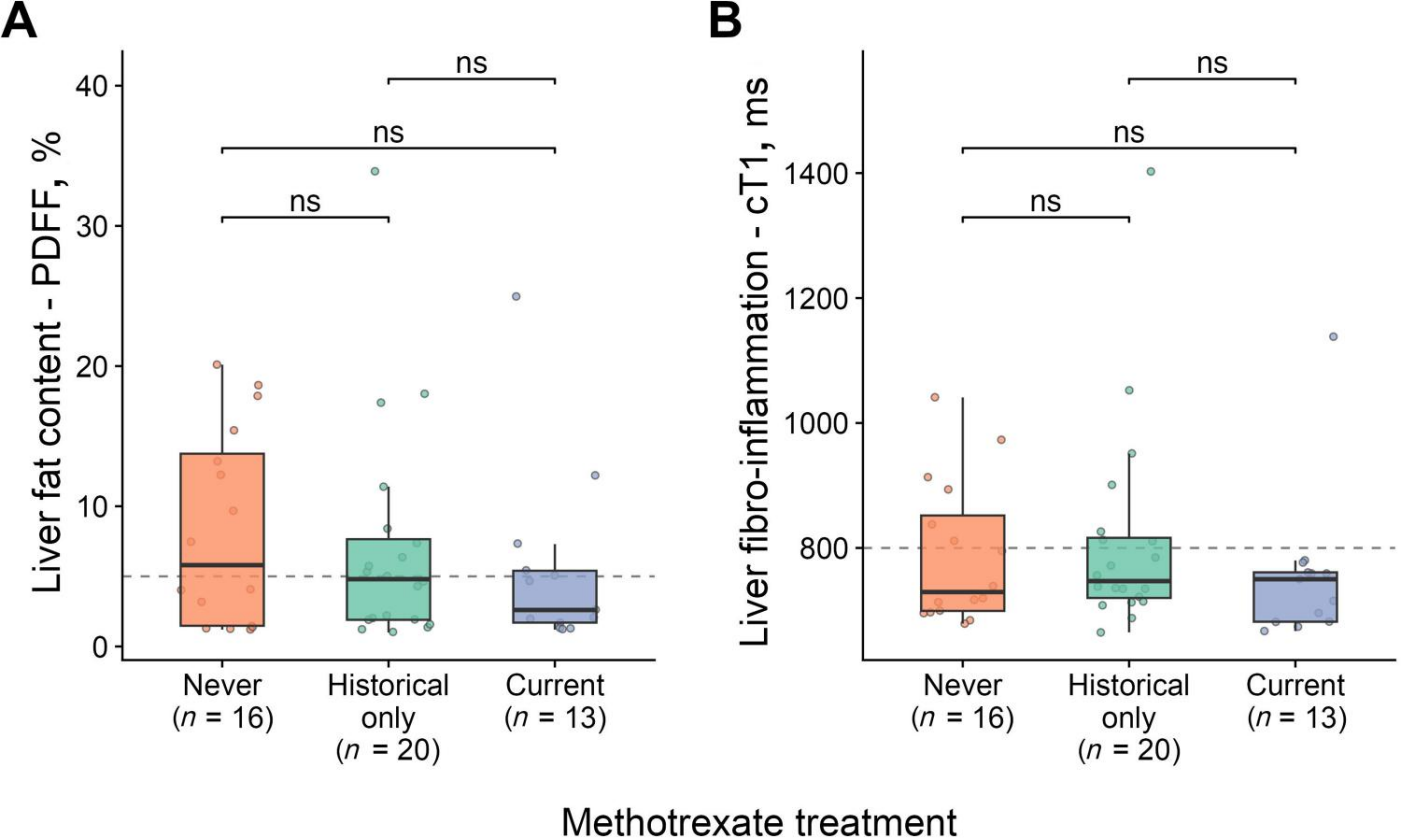
Impact of methotrexate (MTX) use on liver fat and fibro-inflammation in MASLD. (**A**) Distribution of liver fat content measured by proton density fat fraction (PDFF, %) according to MTX use. (**B**) Distribution of liver fibro-inflammation levels measured by corrected T1 (cT1) values according to MTX use. MASLD: metabolic dysfunction-associated steatotic liver disease; ns: not significant

MTX use was common in this cohort, with 13/50 individuals (26%) receiving the treatment at the time of clinical assessment and 31/50 (62%) reporting prior usage at some point during their disease course (mainly individuals with PsA; Supplementary Table S1, available at *Rheumatology* online). However, we found no associations of hepatoxicity, since all liver imaging and biochemical measures were comparable between individuals with and without MTX usage (Fig. 3, Supplementary Table S5, available at *Rheumatology* online).

## Discussion

This is the first study to accurately measure prevalence of liver disease in both PsO and PsA in UK patients from secondary care and the general population, using multiparametric MRI. There were three key findings. We found significantly higher prevalence of metabolic liver disease, MASLD/MetALD and MASH, in people with PsD compared with matched controls and healthy individuals. Secondly, half of PsD patients with metabolic liver disease had normal transaminases, and two-thirds of those with MASH had normal ELF scores. Thirdly, we found no obvious evidence of an association between previous methotrexate use and metabolic liver disease. Instead, the severity of liver disease activity, mainly liver fibro-inflammation, was associated with severity of PsO. Together these findings indicate that MASLD and MetALD are overlooked co-morbidities in PsD and that management of PsD should include screening for these metabolic diseases, rather than for hepatotoxicity alone. Use of multiparametric MRI in PsD will enable earlier detection of concomitant liver disease activity and allow for appropriate management before MASLD/MASH progress to advanced stages.

These findings align with previous works demonstrating that individuals with PsO are at higher risk of MASLD and liver fat deposition [2–6], based on VCTE. This is the first study to extend this association to PsA and to use advanced imaging for liver health characterization. In the UK Biobank PsD participants, liver disease prevalence was lower (33%) compared with our prospective secondary care PsD cohort, despite a higher rate of comorbidities. This may suggest less severe PsD in the UK Biobank, that is closer to the estimated prevalence of MASLD worldwide (30%) [36–38].

Our data suggest that while obesity is a contributing risk factor to MASLD/MetALD in people with PsD, it is not the sole cause. The association across inflammatory disorders driven by similar systemic mediators is well described [39] and is implicated here. We observed a significant increase in liver fibro-inflammation in PsD, independent of liver fat, and elevated liver fibro-inflammation with increased PASI score in PsO, not seen for liver fat.

MTX is the most common routine therapy for PsD. Here we find that the liver disease was not associated with either current or past MTX use, even in people with advanced MASH, where significant liver fibrosis would be expected [40, 41]. These findings are consistent with VCTE literature on individuals with PsO [7, 42, 43] or with rheumatoid arthritis [44], where risk of fibrosis was associated with increased metabolic risk, not with MTX use.

Current PsD guidelines recommend routine liver biochemistry to detect early signs of drug-induced liver injury. We found half of the individuals with PsD and MASLD/MetALD showed normal routine blood biochemistry despite having liver abnormalities on imaging, highlighting potential under-recognition and missed diagnoses. Herein the ELF test missed over half of individuals with MASH or advanced MASH. Prevalence of advanced fibrosis defined by ELF test results was low (9%), and as reported in existing literature for PsO [7, 43]. This test has been granted *de novo* authorization as a prognostic tool to aid in assessment of MASH disease progression [45]. Furthermore, unlike with cT1 there was no increase in ELF with increasing PsD severity, suggesting that this test for advanced fibrosis does not provide insight into inflammatory comorbidity in the liver of patients with PsD. The degree of systemic inflammation in PsD could confound ELF as a blood-based marker, as observed for rheumatoid arthritis [44].

Implementing sequential non-invasive tests to screen patients with type 2 diabetes or obesity for high-risk MASLD leads to reduced long-term healthcare costs [46, 47]. Importantly, a multinational randomized controlled trial demonstrated that adding multiparametric MRI to standard care for adults with suspected MASLD is both cost-effective and improves diagnostic rates [29]. Although cost-effectiveness has not yet been established in populations with PsD, screening may still be warranted given our findings, particularly targeted screening in selected groups. Given the current challenges of existing tests, and the typically asymptomatic nature of early MASH, there is a need for inclusion of more sensitive tests, such as multiparametric MRI for (targeted) screening, irrespective of treatment course for PsD, after careful consideration of referral pathways.

One of the strengths of our study was that recruitment from real-world rheumatology and dermatology clinics provided a representative diverse sample of patients, with clinician- and patient-reported disease activity scores, that was validated by individuals with PsD from the general population. By incorporating MRI biomarkers of liver fat and fibro-inflammation, beyond serological markers, we contribute with reliable evidence on MASLD comorbidity in PsD. The study was powered adequately to observe prevalence differences between included populations. Nevertheless, the individuals recruited from specialist centres were relatively few, as were individuals with PsO, who were not examined by a rheumatologist for joint disease, although signs or symptoms of arthritis were confirmed by their dermatologists. There may have been selection bias by capturing participants from secondary care with more severe disease. While the substantial number of matched and healthy controls enabled a robust comparative assessment, some differences remained, as UK Biobank participants were older with more comorbidities.

There may be some limitations on the generalizability of our findings and in particular, ethnicity data were not collected in the COLIPSO study. As a real-world study, treatment decisions will also have been influenced by individual patient attributes. Cumulative MTX dosage data were unavailable for individuals with prior exposure, as information on treatment duration and start/stop dates was not recorded. This limits our ability to assess the long-term hepatotoxic impact of MTX. However, the prevalence of MASLD and MASH was not associated with the duration of PsD (either PsA or PsO), suggesting that other factors, such as metabolic comorbidities, may play a more prominent role in liver involvement. The systemic inflammatory nature of PsD, alongside normal biochemistry, raises the question of whether other organ abnormalities may be missed by reliance on blood markers alone. Future research in PsD should explore the utility of multi-organ imaging markers, alongside other serological markers to examine the inflammatory burden in other organs.

## Conclusion

This study highlights the complex interplay between MASLD and psoriatic disease, emphasizing the need for early detection with multiparametric MRI. This approach will enable a more accurate assessment of disease burden, which will facilitate effective management, and support ongoing monitoring of high-risk individuals to improve their outcomes.

## Supplementary Material

keaf344_Supplementary_Data

## Data Availability

Summary data are included in the manuscript or uploaded as online supplemental information. Anonymized individual patient data can be shared upon request or as required by law and/or regulation and/or governance by and within the rules of UK Biobank access with qualified external researchers. Approval of such requests is at the discretion of the study sponsors and is dependent on the nature of the request, the merit of the research proposed, the availability of the data and the intended use of the data.
