# Supplementary material for: High prevalence of MASLD in psoriasis and psoriatic arthritis assessed with multiparametric magnetic resonance imaging
Source: Rheumatology (Oxford). 2025 Jun 26;64(11):5741–50. doi: 10.1093/rheumatology/keaf344 (PMC12596067; doi:10.1093/rheumatology/keaf344)
Supplement: keaf344_Supplementary_Data [file keaf344_supplementary_data.docx]

***Title:*** High prevalence of MASLD in Psoriasis and Psoriatic Arthritis assessed with multiparametric magnetic resonance imaging

***Supplementary tables and figures***

|  | **Legend** | **Page** |
| --- | --- | --- |
| **Supplementary Data S1** | Methodology | 2 |
| **Supplementary Data S2** | Patient reported outcomes and patient self-reported outcomes related to health-related quality of life | 3 |
| **Supplementary Figure S1** | Study population | 4 |
| **Supplementary Table S1** | Demographic, clinical, biochemical and MRI characteristics of PsD population. | 6 |
| **Supplementary Table S2** | Comparison of characteristics between PsD individuals with and without MASLD/MetALD. | 10 |
| **Supplementary Table S3** | Comparison of characteristics between PsD individuals with and without MASH. | 12 |
| **Supplementary Table S4** | Spearman’s rank correlation coefficient between liver fat content (PDFF), liver inflammation (cT1) and psoriatic disease severity measures. | 14 |
| **Supplementary Table S5** | Comparison of characteristics between PsD individuals with and without MTX usage. | 15 |
| **Supplementary References** | | 17 |

***Supplementary Data S1***

***Control group matching***

Matched controls were selected using propensity score matching to minimise confounding. We applied 1:3 case-to-control nearest-neighbour matching, pairing each PsD individual with three non-psoriatic controls based on the matching criteria. For metabolic matched controls this was based on age, sex, BMI, hypertension, hyperlipidaemia and diabetes status. For age and sex matched controls, these were the only 2 criteria. The matching targeted the Average Treatment Effect on the Treated (ATT) to balance covariates between groups. Propensity scores were calculated using the ‘MatchIt’ package in R (1).

***Psoriatic disease ICD-10 codes***

We identified individuals from the UK Biobank with PsD using the following ICD-10 codes:

PsA: M07.0, M07.1, M07.2, M07.3, L40.5, M09.0

PsO: L40.0, L40.1, L40.2, L40.3, L40.4, L40.8, L40.9

**Blood tests**

Abnormality was defined based on local and laboratory clinical recommendations:

- CRP ≥5 mg/L
- Bilirubin ≥21 µmol/L
- Albumin ≥50 g/L
- ALT ≥50 IU/L [Male] or ≥35 IU/L [Female]
- ALP ≥130 IU/L
- ELF ≥9.8

***Supplementary Data S2***

**Patient self-reported outcomes related to health-related quality of life and pain scores**

**Psoriatic Arthritis Impact of Disease-12 (PsAID-12)**

The PsAID-12 is a validated 12-item questionnaire scored from 0 to 10, assessing pain, fatigue, skin issues, daily activities, function, discomfort, sleep, coping, anxiety, embarrassment, social participation, and depression. The score assesses the impact of disease, and higher scores indicate worse patient-reported outcomes. (2)

**Health Assessment Questionnaire Disability Index (HAQ-DI)**
The HAQ-DI measures functional ability in individuals with rheumatic diseases, including PsA. Scores are categorised as follows: <0.8 (mild to moderate difficulty), 0.8 to <1.2 (moderate to severe disability), and ≥1.2 (severe to very severe disability). (3)

**Dermatology Life Quality Index (DLQI)**
The DLQI is a validated 10-item questionnaire designed to assess the impact of skin conditions (psoriasis) on QoL over the past week. Impact categories are as follows: 0–1 (none), 2–5 (small), 6–10 (moderate), 11–20 (very large), and 21–30 (extremely large). Higher scores indicate a greater impact on QoL. (4)

**36-Item Short Form Survey (SF-36)**
 The SF-36 is a widely used health related QoL measure across various conditions. It includes 36 questions assessing eight health domains, with scores ranging from 0 (maximum impairment) to 100 (no impairment). Results are summarised into two components: Physical (PCS) and Mental (MCS). (5)

**Patient Questionnaire 4 - VAS Scales**
The Patient Questionnaire 4 uses four Visual Analog Scales (VAS) to assess the impact of psoriasis and arthritis on a patient's well-being over the past week. Each question is scored on a 0–10 scale, with higher scores indicating worse outcomes. The four assessed domains are:

1. Overall impact of psoriasis and arthritis (0 = Very good; 10 = Very bad)
2. Pain severity related to psoriatic arthritis (0 = None; 10 = Very severe)
3. Impact of psoriasis alone (0 = Very good; 10 = Very bad)
4. Impact of arthritis alone (0 = Not active; 10 = Very active)

These patient-reported outcomes provide a comprehensive evaluation of symptom severity and disease impact. (6)

**Supplementary Figure S1:** Study population.


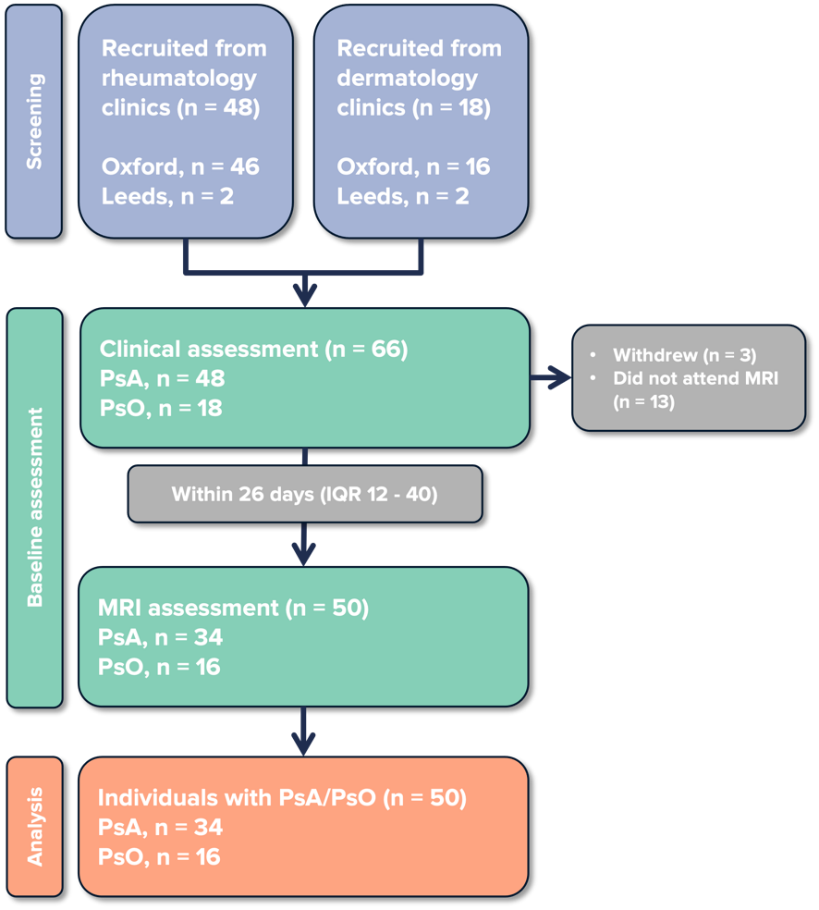


**Supplementary Table S1:** Demographic, clinical, biochemical and MRI characteristics of prospective PsD population from COLIPSO cohort. Text in bold indicate statistical significance of p-value of ≤0.05.

|  | N | PsD  (n = 50) | PsA  (n = 34) | PsO  (n = 16) | P-value |
| --- | --- | --- | --- | --- | --- |
| Baseline Demographics | | | | | |
| Age (years) [mean (SD)] | 50 | 48 (14) | 48 (14) | 47 (14) | 0.884 |
| Sex (Male) [n (%)] | 50 | 29 (58%) | 17 (50%) | 12 (75%) | 0.129 |
| BMI (kg/m^2^) [mean (SD)] | 50 | 29 (5) | 29 (5) | 29 (6) | 0.655 |
| Categories [n (%)] | 50 |  |  |  | >0.999 |
| Lean (< 25kg/m^2^) |  | 10 (20%) | 7 (21%) | 3 (19%) |  |
| Overweight (≥ 25 - < 30kg/m^2^) |  | 24 (48%) | 16 (47%) | 8 (50%) |  |
| Obese (≥ 30 kg/m^2^) |  | 16 (32%) | 11 (32%) | 5 (31%) |  |
| Type 2 Diabetes [n (%)] | 50 | 1 (2%) | 1 (3%) | 0 (0%) | >0.999 |
| Type 1 Diabetes [n (%)] | 50 | 2 (4%) | 1 (3%) | 1 (6%) | 0.542 |
| Hypertension [n (%)] | 50 | 10 (20%) | 10 (29%) | 0 (0%) | **0.02** |
| Hyperlipidaemia [n (%)] | 50 | 9 (18%) | 6 (18%) | 3 (19%) | >0.999 |
| Cardiometabolic risk factor [n (%)] | 50 | 43 (86%) | 30 (88%) | 13 (81%) | 0.666 |
| High alcohol use [n (%)] | 50 | 3 (6%) | 1 (3%) | 2 (13%) | 0.237 |
| SF-36 health survey | | | | | |
| Physical functioning (%) [mean (SD)] | 48 | 58 (26) | 51 (23) | 73 (25) | **0.006** |
| Role limitations due to physical health (%) [mean (SD)] | 48 | 39 (41) | 32 (37) | 57 (44) | **0.041** |
| Role limitations due to emotional problems (%) [mean (SD)] | 48 | 55 (44) | 51 (45) | 64 (42) | 0.378 |
| Energy/fatigue (%) [mean (SD)] | 48 | 38 (22) | 37 (23) | 42 (17) | 0.413 |
| Emotional well-being (%) [mean (SD)] | 48 | 64 (19) | 64 (20) | 62 (17) | 0.665 |
| Social functioning (%) [mean (SD)] | 48 | 63 (24) | 61 (23) | 69 (27) | 0.264 |
| Pain (%) [mean (SD)] | 48 | 46 (25) | 40 (18) | 58 (33) | **0.042** |
| General health (%) [mean (SD)] | 48 | 40 (22) | 35 (21) | 54 (20) | **0.006** |
| PsA/PsO characteristics | | | | | |
| Duration skin condition (years) [mean (SD)] | 50 | 19 (15) | 20 (15) | 17 (15) | 0.581 |
| PASI score [mean (SD)] | 50 | 4.3 (7) | 1.5 (2) | 10.1 (9) | **<0.001** |
| Categories [n (%)] | 50 |  |  |  | **<0.001** |
| Mild (<5) |  | 36 (72%) | 32 (94%) | 4 (25%) |  |
| Moderate (5-10) |  | 6 (12%) | 2 (6%) | 4 (25%) |  |
| Severe (≥10) |  | 8 (16%) | 0 (0%) | 8 (50%) |  |
| BSA coverage (%) [mean (SD)] | 50 | 7.3 (12) | 2.0 (2) | 18.4 (15) | **<0.001** |
| Categories [n (%)] | 50 |  |  |  | **<0.001** |
| Mild (<5) |  | 32 (64%) | 29 (85%) | 3 (19%) |  |
| Moderate (5-10) |  | 8 (16%) | 5 (15%) | 3 (19%) |  |
| Severe (≥10) |  | 10 (20%) | 0 (0%) | 10 (63%) |  |
| PsA only measures | | | | | |
| Duration PsA (years) [mean (SD)] | 36 | 10 (10) | 10 (10) | - | - |
| DAPSA score [mean (SD)] | 35 | 33 (23) | 33 (23) | - | - |
| Categories [n (%)] | 35 |  |  |  |  |
| Remission (<5) |  | 0 (0%) | 0 (0%) |  |  |
| Low (5-15) |  | 7 (20%) | 6 (18%) |  |  |
| Moderate (15-28) |  | 12 (34%) | 12 (35%) |  |  |
| High (≥28) |  | 16 (46%) | 16 (47%) |  |  |
| Treatment | | | | | |
| MTX treatment group [n (%)] | 49 |  |  |  | **<0.001** |
| Never |  | 16 (33%) | 5 (15%) | 11 (69%) |  |
| Historical only |  | 20 (41%) | 16 (48%) | 4 (25%) |  |
| Current |  | 13 (27%) | 12 (36%) | 1 (6%) |  |
| Biochemical | | | | | |
| C-reactive protein (mg/L) [mean (SD)] | 47 | 6.7 (12) | 7.7 (14) | 4.3 (4) | 0.821 |
| CRP (≥5 mg/L) [n (%)] | 47 | 17 (36%) | 14 (41%) | 3 (23%) | 0.321 |
| Bilirubin (µmol/L) [mean (SD)] | 50 | 11.0 (6) | 10.7 (5) | 11.8 (9) | 0.654 |
| Bilirubin (≥21 µmol/L) [n (%)] | 50 | 4 (8%) | 2 (6%) | 2 (13%) | 0.584 |
| Albumin (g/L) [mean (SD)] | 50 | 41 (4) | 41 (4) | 42 (4) | 0.195 |
| Albumin (≥50 g/L) [n (%)] | 50 | 0 (0%) | 0 (0%) | 0 (0%) | - |
| ALT (IU/L) [mean (SD)] | 50 | 33 (24) | 27 (16) | 44 (33) | **0.029** |
| ALT (Male ≥50 IU/L, Female ≥35 IU/L) [n (%)] | 50 | 10 (20%) | 7 (21%) | 3 (19%) | >0.999 |
| ALP (IU/L) [mean (SD)] | 50 | 81 (23) | 84 (20) | 74 (28) | 0.303 |
| ALP (≥130 IU/L) [n (%)] | 50 | 2 (4%) | 2 (6%) | 0 (0%) | >0.999 |
| ELF [mean (SD)] | 47 | 8.7 (1) | 8.8 (1) | 8.7 (1) | 0.6 |
| ELF (≥9.8) [n (%)] | 47 | 4 (9%) | 3 (9%) | 1 (7%) | >0.999 |
| Baseline MRI Liver Metrics | | | | | |
| Liver fat content - PDFF (%) [mean (SD)] | 50 | 7.2 (7) | 6.5 (8) | 8.8 (6) | 0.116 |
| PDFF ≥ 5% [n (%)] | 50 | 24 (48%) | 15 (44%) | 9 (56%) | 0.547 |
| MASLD/MetALD (PDFF ≥ 5% & metabolic risk factor) [n (%)] | 50 | 22 (44%) | 14 (41%) | 8 (50%) | 0.761 |
| Liver fibro-inflammation - cT1 (ms) [mean (SD)] | 50 | 790 (138) | 790 (146) | 792 (124) | 0.811 |
| cT1 ≥ 800ms [n (%)] | 50 | 15 (30%) | 9 (26%) | 6 (38%) | 0.514 |
| cT1 ≥ 875ms [n (%)] | 50 | 9 (18%) | 4 (12%) | 5 (31%) | 0.124 |
| Steatohepatitis (cT1 ≥ 800ms & PDFF ≥ 5%) [n (%)] | 50 | 12 (24%) | 6 (18%) | 6 (38%) | 0.163 |
| MASH (cT1 ≥ 800ms & PDFF ≥ 5% & metabolic risk factor) [n (%)] | 50 | 11 (22%) | 6 (18%) | 5 (31%) | 0.297 |
| Severe MASH (cT1 ≥ 875ms & PDFF ≥ 5% & cardiometabolic risk factor) [n (%)] |  |  |  |  |  |

**Supplementary Table S2:** Comparison of characteristics between PsD individuals with and without MASLD/MetALD. Text in bold indicate statistical significance of p-value of ≤0.05.

|  | N | Normal liver fat content  (n = 28) | MASLD/MetALD  (n = 22) | P-value |
| --- | --- | --- | --- | --- |
| Baseline Demographics | | | | |
| Age (years) [mean (SD)] | 50 | 44 (14) | 52 (12) | **0.015** |
| Sex (Male) [n (%)] | 50 | 14 (50%) | 15 (68%) | 0.254 |
| BMI (kg/m^2^) [mean (SD)] | 50 | 27 (3) | 31 (6) | **0.009** |
| Categories [n (%)] | 50 |  |  | **0.042** |
| Lean (< 25kg/m^2^) |  | 8 (29%) | 2 (9%) |  |
| Overweight (≥ 25 - < 30kg/m^2^) |  | 15 (54%) | 9 (41%) |  |
| Obese (≥ 30 kg/m^2^) |  | 5 (18%) | 11 (50%) |  |
| PsA [n (%)] | 50 | 20 (71%) | 14 (64%) | 0.761 |
| PsO [n (%)] | 50 | 8 (29%) | 8 (36%) | 0.761 |
| Type 2 Diabetes [n (%)] | 50 | 0 (0%) | 1 (5%) | 0.44 |
| Type 1 Diabetes [n (%)] | 50 | 1 (4%) | 1 (5%) | >0.999 |
| Hypertension [n (%)] | 50 | 4 (14%) | 6 (27%) | 0.302 |
| Hyperlipidaemia [n (%)] | 50 | 3 (11%) | 6 (27%) | 0.157 |
| Cardiometabolic risk factor [n (%)] | 50 | 21 (75%) | 22 (100%) | **0.014** |
| High alcohol use [n (%)] | 50 | 0 (0%) | 3 (14%) | 0.079 |
| SF-36 questionnaire | | | | |
| Physical functioning (%) [mean (SD)] | 48 | 58 (25) | 57 (27) | 0.802 |
| Role limitations due to physical health (%) [mean (SD)] | 48 | 37 (38) | 42 (44) | 0.861 |
| Role limitations due to emotional problems (%) [mean (SD)] | 48 | 53 (43) | 57 (47) | 0.698 |
| Energy/fatigue (%) [mean (SD)] | 48 | 37 (19) | 40 (25) | 0.909 |
| Emotional well-being (%) [mean (SD)] | 48 | 61 (18) | 67 (20) | 0.471 |
| Social functioning (%) [mean (SD)] | 48 | 62 (24) | 65 (25) | 0.454 |
| Pain (%) [mean (SD)] | 48 | 48 (24) | 43 (26) | 0.477 |
| General health (%) [mean (SD)] | 48 | 43 (23) | 37 (21) | 0.348 |
| PsA/PsO characteristics | | | | |
| Duration skin condition (years) [mean (SD)] | 50 | 21 (14) | 17 (15) | 0.129 |
| PASI score [mean (SD)] | 50 | 3.3 (3) | 5.6 (9) | 0.984 |
| Categories [n (%)] | 50 |  |  | 0.492 |
| Mild (<5) |  | 22 (79%) | 14 (64%) |  |
| Moderate (5-10) |  | 3 (11%) | 3 (14%) |  |
| Severe (≥10) |  | 3 (11%) | 5 (23%) |  |
| BSA coverage (%) [mean (SD)] | 50 | 6.1 (9) | 8.8 (15) | 0.811 |
| Categories [n (%)] | 50 |  |  | 0.922 |
| Mild (<5) |  | 18 (64%) | 14 (64%) |  |
| Moderate (5-10) |  | 5 (18%) | 3 (14%) |  |
| Severe (≥10) |  | 5 (18%) | 5 (23%) |  |
| PsA only measures | | | | |
| Duration PsA (years) [mean (SD)] | 36 | 12 (12) | 7 (5) | 0.469 |
| DAPSA score [mean (SD)] | 35 | 32 (19) | 35 (28) | >0.999 |
| Categories [n (%)] | 35 |  |  | 0.737 |
| Remission (<5) |  | 0 (0%) | 0 (0%) |  |
| Low (5-15) |  | 5 (24%) | 2 (14%) |  |
| Moderate (15-28) |  | 6 (29%) | 6 (43%) |  |
| High (≥28) |  | 10 (48%) | 6 (43%) |  |
| Treatment | | | | |
| MTX treatment group [n (%)] | 49 |  |  | >0.999 |
| Never |  | 9 (32%) | 7 (33%) |  |
| Historical only |  | 11 (39%) | 9 (43%) |  |
| Current |  | 8 (29%) | 5 (24%) |  |
| Biochemical | | | | |
| C-reactive protein (mg/L) [mean (SD)] | 47 | 6.7 (13) | 6.8 (12) | 0.644 |
| CRP (≥5 mg/L) [n (%)] | 47 | 9 (33%) | 8 (40%) | 0.761 |
| Bilirubin (µmol/L) [mean (SD)] | 50 | 9.9 (5) | 12.4 (8) | 0.367 |
| Bilirubin (≥21 µmol/L) [n (%)] | 50 | 1 (4%) | 3 (14%) | 0.308 |
| Albumin (g/L) [mean (SD)] | 50 | 42 (4) | 41 (4) | 0.937 |
| Albumin (≥50 g/L) [n (%)] | 50 | 0 (0%) | 0 (0%) | - |
| ALT (IU/L) [mean (SD)] | 50 | 28 (25) | 38 (21) | **0.021** |
| ALT (Male ≥50 IU/L, Female ≥35 IU/L) [n (%)] | 50 | 3 (11%) | 7 (32%) | 0.084 |
| ALP (IU/L) [mean (SD)] | 50 | 77 (25) | 86 (20) | 0.174 |
| ALP (≥130 IU/L) [n (%)] | 50 | 1 (4%) | 1 (5%) | >0.999 |
| ELF [mean (SD)] | 47 | 8.5 (1) | 9.1 (1) | **0.02** |
| ELF (≥9.8) [n (%)] | 47 | 0 (0%) | 4 (19%) | **0.034** |
| Baseline MRI Liver Metrics | | | | |
| Liver fat content - PDFF (%) [mean (SD)] | 50 | 2.8 (2) | 12.9 (8) | **<0.001** |
| PDFF ≥ 5% [n (%)] | 50 | 2 (7%) | 22 (100%) | **<0.001** |
| MASLD/MetALD (PDFF ≥ 5% & metabolic risk factor) [n (%)] | 50 |  |  |  |
| Liver fibro-inflammation - cT1 (ms) [mean (SD)] | 50 | 741 (55) | 853 (183) | **0.027** |
| cT1 ≥ 800ms [n (%)] | 50 | 4 (14%) | 11 (50%) | **0.012** |
| cT1 ≥ 875ms [n (%)] | 50 | 1 (4%) | 8 (36%) | **0.007** |
| Steatohepatitis (cT1 ≥ 800ms & PDFF ≥ 5%) [n (%)] | 50 | 1 (4%) | 11 (50%) | **<0.001** |
| MASH (cT1 ≥ 800ms & PDFF ≥ 5% & metabolic risk factor) [n (%)] | 50 | 0 (0%) | 11 (50%) | **<0.001** |
| Severe MASH (cT1 ≥ 875ms & PDFF ≥ 5% & cardiometabolic risk factor) [n (%)] | 50 | 0 (0%) | 8 (36%) | **<0.001** |

**Supplementary Table S3:** Comparison of characteristics between PsD individuals with and without MASH. Text in bold indicate statistical significance of p-value of ≤0.05.

|  | N | No MASH  (n = 39) | MASH  (n = 11) | P-value |
| --- | --- | --- | --- | --- |
| Baseline Demographics | | | | |
| Age (years) [mean (SD)] | 50 | 47 (14) | 50 (11) | 0.399 |
| Sex (Male) [n (%)] | 50 | 21 (54%) | 8 (73%) | 0.319 |
| BMI (kg/m^2^) [mean (SD)] | 50 | 28 (4) | 32 (7) | **0.036** |
| Categories [n (%)] | 50 |  |  | 0.088 |
| Lean (< 25kg/m^2^) |  | 10 (26%) | 0 (0%) |  |
| Overweight (≥ 25 - < 30kg/m^2^) |  | 19 (49%) | 5 (45%) |  |
| Obese (≥ 30 kg/m^2^) |  | 10 (26%) | 6 (55%) |  |
| PsA [n (%)] | 50 | 28 (72%) | 6 (55%) | 0.297 |
| PsO [n (%)] | 50 | 11 (28%) | 5 (45%) | 0.297 |
| Type 2 Diabetes [n (%)] | 50 | 1 (3%) | 0 (0%) | >0.999 |
| Type 1 Diabetes [n (%)] | 50 | 1 (3%) | 1 (9%) | 0.395 |
| Hypertension [n (%)] | 50 | 7 (18%) | 3 (27%) | 0.671 |
| Hyperlipidaemia [n (%)] | 50 | 7 (18%) | 2 (18%) | >0.999 |
| Cardiometabolic risk factor [n (%)] | 50 | 32 (82%) | 11 (100%) | 0.324 |
| High alcohol use [n (%)] | 50 | 1 (3%) | 2 (18%) | 0.118 |
| SF-36 questionnaire | | | | |
| Physical functioning (%) [mean (SD)] | 48 | 58 (26) | 56 (26) | 0.929 |
| Role limitations due to physical health (%) [mean (SD)] | 48 | 37 (38) | 48 (51) | 0.584 |
| Role limitations due to emotional problems (%) [mean (SD)] | 48 | 51 (43) | 70 (48) | 0.229 |
| Energy/fatigue (%) [mean (SD)] | 48 | 38 (23) | 38 (18) | 0.859 |
| Emotional well-being (%) [mean (SD)] | 48 | 63 (19) | 67 (16) | 0.592 |
| Social functioning (%) [mean (SD)] | 48 | 63 (25) | 64 (23) | 0.908 |
| Pain (%) [mean (SD)] | 48 | 48 (23) | 39 (30) | 0.251 |
| General health (%) [mean (SD)] | 48 | 42 (22) | 35 (24) | 0.438 |
| PsA/PsO characteristics | | | | |
| Duration skin condition (years) [mean (SD)] | 50 | 20 (14) | 17 (17) | 0.307 |
| PASI score [mean (SD)] | 50 | 2.8 (3) | 9.4 (12) | 0.096 |
| Categories [n (%)] | 50 |  |  | **0.005** |
| Mild (<5) |  | 32 (82%) | 4 (36%) |  |
| Moderate (5-10) |  | 4 (10%) | 2 (18%) |  |
| Severe (≥10) |  | 3 (8%) | 5 (45%) |  |
| BSA coverage (%) [mean (SD)] | 50 | 5.6 (9) | 13.3 (18) | 0.139 |
| Categories [n (%)] | 50 |  |  | 0.222 |
| Mild (<5) |  | 27 (69%) | 5 (45%) |  |
| Moderate (5-10) |  | 6 (15%) | 2 (18%) |  |
| Severe (≥10) |  | 6 (15%) | 4 (36%) |  |
| PsA only measures | | | | |
| Duration PsA (years) [mean (SD)] | 36 | 10 (10) | 8 (6) | 0.766 |
| DAPSA score [mean (SD)] | 35 | 33 (23) | 33 (25) | 0.948 |
| Categories [n (%)] | 35 |  |  | 0.248 |
| Remission (<5) |  | 0 (0%) | 0 (0%) |  |
| Low (5-15) |  | 7 (24%) | 0 (0%) |  |
| Moderate (15-28) |  | 8 (28%) | 4 (67%) |  |
| High (≥28) |  | 14 (48%) | 2 (33%) |  |
| Treatment | | | | |
| MTX treatment group [n (%)] | 49 |  |  | 0.326 |
| Never |  | 11 (28%) | 5 (50%) |  |
| Historical only |  | 16 (41%) | 4 (40%) |  |
| Current |  | 12 (31%) | 1 (10%) |  |
| Biochemical | | | | |
| C-reactive protein (mg/L) [mean (SD)] | 47 | 7.0 (14) | 5.6 (4) | 0.264 |
| CRP (≥5 mg/L) [n (%)] | 47 | 12 (32%) | 5 (50%) | 0.46 |
| Bilirubin (µmol/L) [mean (SD)] | 50 | 10.1 (5) | 14.4 (8) | 0.076 |
| Bilirubin (≥21 µmol/L) [n (%)] | 50 | 2 (5%) | 2 (18%) | 0.206 |
| Albumin (g/L) [mean (SD)] | 50 | 41 (4) | 41 (4) | >0.999 |
| Albumin (≥50 g/L) [n (%)] |  |  |  |  |
| ALT (IU/L) [mean (SD)] | 50 | 28 (22) | 47 (24) | **0.004** |
| ALT (Male ≥50 IU/L, Female ≥35 IU/L) [n (%)] | 50 | 5 (13%) | 5 (45%) | **0.03** |
| ALP (IU/L) [mean (SD)] | 50 | 80 (24) | 86 (19) | 0.228 |
| ALP (≥130 IU/L) [n (%)] | 50 | 2 (5%) | 0 (0%) | >0.999 |
| ELF [mean (SD)] | 47 | 8.6 (1) | 9.1 (1) | 0.191 |
| ELF (≥9.8) [n (%)] | 47 | 1 (3%) | 3 (27%) | **0.035** |
| Baseline MRI Liver Metrics | | | | |
| Liver fat content - PDFF (%) [mean (SD)] | 50 | 4.1 (3) | 18.4 (7) | **<0.001** |
| PDFF ≥ 5% [n (%)] | 50 | 13 (33%) | 11 (100%) | **<0.001** |
| MASLD/MetALD (PDFF ≥ 5% & metabolic risk factor) [n (%)] | 50 | 11 (28%) | 11 (100%) | **<0.001** |
| Liver fibro-inflammation - cT1 (ms) [mean (SD)] | 50 | 736 (52) | 985 (173) | **<0.001** |
| cT1 ≥ 800ms [n (%)] | 50 | 4 (10%) | 11 (100%) | **<0.001** |
| cT1 ≥ 875ms [n (%)] | 50 | 1 (3%) | 8 (73%) | **<0.001** |
| Steatohepatitis (cT1 ≥ 800ms & PDFF ≥ 5%) [n (%)] | 50 | 1 (3%) | 11 (100%) | **<0.001** |
| MASH (cT1 ≥ 800ms & PDFF ≥ 5% & metabolic risk factor) [n (%)] | 50 | 0 (0%) | 11 (11%) | **<0.001** |
| Severe MASH (cT1 ≥ 875ms & PDFF ≥ 5% & cardiometabolic risk factor) [n (%)] | 50 | 0 (0%) | 8 (73%) | **<0.001** |

**Supplementary Table S4:** Spearman’s rank correlation coefficient between liver fat content (PDFF), liver inflammation (cT1) and psoriatic disease severity measures. * Indicates statistical significance.

|  | Liver fat content – PDFF (%) | | | Liver fibro-inflammation – cT1 (ms) | | |
| --- | --- | --- | --- | --- | --- | --- |
| Disease severity Metric | PsA only  (n = 34) | PsO only  (n = 16) | PsA & PsO  (n = 50) | PsA only  (n = 34) | PsO only  (n = 16) | PsA & PsO  (n = 50) |
| Psoriasis disease |  |  |  |  |  |  |
| Duration skin condition (years) | -0.284 | 0.071 | -0.212 | -0.057 | -0.068 | -0.067 |
| PASI score | -0.120 | 0.472 | 0.159 | 0.081 | **0.582*** | 0.102 |
| BSA coverage (%) | -0.111 | 0.312 | 0.149 | 0.057 | 0.347 | 0.057 |
| Psoriatic arthritis disease |  |  |  |  |  |  |
| Duration arthritis (years) | -0.127 | - | - | -0.122 | - | - |
| DAPSA score | -0.101 | - | - | 0.080 | - | - |
| SF-36 questionnaire |  |  |  |  |  |  |
| Physical functioning (%) | 0.108 | -0.004 | 0.124 | -0.116 | 0.090 | -0.127 |
| Role limitations due to physical health (%) | 0.331 | -0.182 | 0.181 | 0.123 | 0.046 | 0.061 |
| Role limitations due to emotional problems (%) | 0.221 | -0.006 | 0.171 | 0.069 | 0.253 | 0.125 |
| Energy/fatigue (%) | 0.132 | 0.221 | 0.142 | -0.160 | 0.446 | 0.000 |
| Emotional well-being (%) | 0.155 | 0.276 | 0.141 | -0.141 | 0.260 | 0.008 |
| Social functioning (%) | 0.333 | -0.388 | 0.107 | -0.056 | -0.199 | -0.158 |
| Pain (%) | -0.102 | -0.166 | -0.137 | -0.210 | 0.076 | -0.215 |
| General health (%) | 0.120 | -0.066 | 0.002 | -0.069 | 0.168 | -0.023 |

**Supplementary Table S5:** Comparison of characteristics between PsD individuals with and without MTX usage. Text in bold indicate statistical significance of p-value of ≤0.05.

|  | N | Current or historic MTX treatment  (n = 33) | No MTX treatment ever  (n = 16) | P-value |
| --- | --- | --- | --- | --- |
| Baseline Demographics | | | | |
| Age (years) [mean (SD)] | 50 | 49 (15) | 47 (12) | 0.757 |
| Sex (Male) [n (%)] | 50 | 20 (61%) | 8 (50%) | 0.547 |
| BMI (kg/m^2^) [mean (SD)] | 50 | 29 (5) | 29 (6) | 0.662 |
| Categories [n (%)] | 50 |  |  | 0.573 |
| Lean (< 25kg/m^2^) |  | 8 (24%) | 2 (13%) |  |
| Overweight (≥ 25 - < 30kg/m^2^) |  | 14 (42%) | 9 (56%) |  |
| Obese (≥ 30 kg/m^2^) |  | 11 (33%) | 5 (31%) |  |
| PsA [n (%)] | 50 | 28 (85%) | 5 (31%) | **<0.001** |
| PsO [n (%)] | 50 | 5 (15%) | 11 (69%) | **<0.001** |
| Type 2 Diabetes [n (%)] | 50 | 1 (3%) | 0 (0%) | >0.999 |
| Type 1 Diabetes [n (%)] | 50 | 0 (0%) | 2 (13%) | 0.102 |
| Hypertension [n (%)] | 50 | 9 (27%) | 1 (6%) | 0.135 |
| Hyperlipidaemia [n (%)] | 50 | 7 (21%) | 2 (13%) | 0.698 |
| Cardiometabolic risk factor [n (%)] | 50 | 28 (85%) | 14 (88%) | >0.999 |
| High alcohol use [n (%)] | 50 | 1 (3%) | 2 (13%) | 0.245 |
| SF-36 questionnaire | | | | |
| Physical functioning (%) [mean (SD)] | 48 | 55 (26) | 65 (23) | 0.162 |
| Role limitations due to physical health (%) [mean (SD)] | 48 | 39 (41) | 43 (41) | 0.715 |
| Role limitations due to emotional problems (%) [mean (SD)] | 48 | 54 (44) | 62 (45) | 0.62 |
| Energy/fatigue (%) [mean (SD)] | 48 | 37 (24) | 42 (17) | 0.428 |
| Emotional well-being (%) [mean (SD)] | 48 | 63 (20) | 65 (17) | 0.851 |
| Social functioning (%) [mean (SD)] | 48 | 65 (22) | 58 (31) | 0.443 |
| Pain (%) [mean (SD)] | 48 | 46 (23) | 44 (29) | 0.717 |
| General health (%) [mean (SD)] | 48 | 37 (22) | 49 (22) | 0.084 |
| PsA/PsO characteristics | | | | |
| Duration skin condition (years) [mean (SD)] | 50 | 19 (15) | 19 (16) | 0.847 |
| PASI score [mean (SD)] | 50 | 2.9 (7) | 7.0 (5) | **<0.001** |
| Categories [n (%)] | 50 |  |  | **0.004** |
| Mild (<5) |  | 29 (88%) | 7 (44%) |  |
| Moderate (5-10) |  | 2 (6%) | 3 (19%) |  |
| Severe (≥10) |  | 2 (6%) | 6 (38%) |  |
| BSA coverage (%) [mean (SD)] | 50 | 4.6 (11) | 12.8 (11) | **<0.001** |
| Categories [n (%)] | 50 |  |  | **<0.001** |
| Mild (<5) |  | 28 (85%) | 4 (25%) |  |
| Moderate (5-10) |  | 2 (6%) | 5 (31%) |  |
| Severe (≥10) |  | 3 (9%) | 7 (44%) |  |
| PsA only measures | | | | |
| Duration PsA (years) [mean (SD)] | 36 | 10 (9) | 13 (15) | >0.999 |
| DAPSA score [mean (SD)] | 35 | 34 (23) | 31 (26) | 0.734 |
| Categories [n (%)] | 35 |  |  | >0.999 |
| Remission (<5) |  | 0 (0%) | 0 (0%) |  |
| Low (5-15) |  | 6 (21%) | 1 (20%) |  |
| Moderate (15-28) |  | 9 (31%) | 2 (40%) |  |
| High (≥28) |  | 14 (48%) | 2 (40%) |  |
| Treatment | | | | |
| MTX treatment group [n (%)] | 49 |  |  | **<0.001** |
| Never |  | 0 (0%) | 16 (100%) |  |
| Historical only |  | 20 (61%) | 0 (0%) |  |
| Current |  | 13 (39%) | 0 (0%) |  |
| Biochemical | | | | |
| C-reactive protein (mg/L) [mean (SD)] | 47 | 7.8 (15) | 3.9 (3) | 0.933 |
| CRP (≥5 mg/L) [n (%)] | 47 | 13 (41%) | 3 (21%) | 0.316 |
| Bilirubin (µmol/L) [mean (SD)] | 50 | 11.1 (6) | 10.4 (7) | 0.404 |
| Bilirubin (≥21 µmol/L) [n (%)] | 50 | 3 (9%) | 1 (6%) | >0.999 |
| Albumin (g/L) [mean (SD)] | 50 | 41 (4) | 42 (4) | 0.202 |
| Albumin (≥50 g/L) [n (%)] | 50 | 0 (0%) | 0 (0%) | - |
| ALT (IU/L) [mean (SD)] | 50 | 31 (19) | 33 (32) | 0.639 |
| ALT (Male ≥50 IU/L, Female ≥35 IU/L) [n (%)] | 50 | 7 (21%) | 2 (13%) | 0.698 |
| ALP (IU/L) [mean (SD)] | 50 | 83 (19) | 76 (30) | 0.424 |
| ALP (≥130 IU/L) [n (%)] | 50 | 2 (6%) | 0 (0%) | >0.999 |
| ELF [mean (SD)] | 47 | 8.9 (1) | 8.5 (1) | 0.166 |
| ELF (≥9.8) [n (%)] | 47 | 4 (13%) | 0 (0%) | 0.298 |
| Baseline MRI Liver Metrics | | | | |
| Liver fat content - PDFF (%) [mean (SD)] | 50 | 6.5 (7) | 8.3 (7) | 0.475 |
| PDFF ≥ 5% [n (%)] | 50 | 15 (45%) | 8 (50%) | >0.999 |
| MASLD/MetALD (PDFF ≥ 5% & metabolic risk factor) [n (%)] | 50 | 14 (42%) | 7 (44%) | >0.999 |
| Liver fibro-inflammation - cT1 (ms) [mean (SD)] | 50 | 790 (152) | 788 (113) | 0.898 |
| cT1 ≥ 800ms [n (%)] | 50 | 8 (24%) | 6 (38%) | 0.501 |
| cT1 ≥ 875ms [n (%)] | 50 | 5 (15%) | 4 (25%) | 0.449 |
| Steatohepatitis (cT1 ≥ 800ms & PDFF ≥ 5%) [n (%)] | 50 | 5 (15%) | 6 (38%) | 0.141 |
| MASH (cT1 ≥ 800ms & PDFF ≥ 5% & metabolic risk factor) [n (%)] | 50 | 5 (15%) | 5 (31%) | 0.261 |
| Severe MASH (cT1 ≥ 875ms & PDFF ≥ 5% & cardiometabolic risk factor) [n (%)] | 50 | 4 (12%) | 4 (25%) | 0.411 |

**References**

1. Rinella ME, Lazarus JV, Ratziu V, Francque SM, Sanyal AJ, Kanwal F, et al. A multisociety Delphi consensus statement on new fatty liver disease nomenclature. J Hepatol. 2023 Dec;79(6):1542–56.
2. Gossec L, de Wit M, Kiltz U, Braun J, Kalyoncu U, Scrivo R, et al. A patient-derived and patient-reported outcome measure for assessing psoriatic arthritis: elaboration and preliminary validation of the Psoriatic Arthritis Impact of Disease (PsAID) questionnaire, a 13-country EULAR initiative. Ann Rheum Dis. 2014;73(6):1012-9.
3. Bruce B, Fries JF. The Stanford Health Assessment Questionnaire: dimensions and practical applications. Health Qual Life Outcomes. 2003;1:20.
4. Finlay AY, Khan GK. Dermatology Life Quality Index (DLQI)--a simple practical measure for routine clinical use. Clin Exp Dermatol. 1994;19(3):210-6.
5. Bruce B, Fries JF. The Stanford Health Assessment Questionnaire: dimensions and practical applications. Health Qual Life Outcomes. 2003;1:20.
6. Ye, W., Hackett, S., Vandevelde, C., Twigg, S., Helliwell, P.S., Coates, L.C et al. Comparing the Visual Analog Scale and the Numerical Rating Scale in Patient-reported Outcomes in Psoriatic Arthritis. The Journal of Rheumatology 2021;48:836–840.
